# A previously unobserved conformation for the human Pex5p receptor suggests roles for intrinsic flexibility and rigid domain motions in ligand binding

**DOI:** 10.1186/1472-6807-7-24

**Published:** 2007-04-11

**Authors:** Will A Stanley, Niko V Pursiainen, Elspeth F Garman, André H Juffer, Matthias Wilmanns, Petri Kursula

**Affiliations:** 1 EMBL-Hamburg, c/o DESY, Notkestraβe 85, 22603 Hamburg, Germany; 2 Centre for Cellular and Molecular Biology, Council of Scientific and Industrial Research, Uppal Road, Hyderabad 500 007, India; 3 Department of Biochemistry, University of Oulu, FIN-90014, Oulu, Finland; 4 Biocenter Oulu, University of Oulu, FIN-90014, Oulu, Finland; 5 Laboratory of Molecular Biophysics, Department of Biochemistry, University of Oxford, Oxford OX1 3QU, UK

## Abstract

**Background:**

The C-terminal tetratricopeptide (TPR) repeat domain of Pex5p recognises proteins carrying a peroxisomal targeting signal type 1 (PTS1) tripeptide in their C-terminus. Previously, structural data have been obtained from the TPR domain of Pex5p in both the liganded and unliganded states, indicating a conformational change taking place upon cargo protein binding. Such a conformational change would be expected to play a major role both during PTS1 protein recognition as well as in cargo release into the peroxisomal lumen. However, little information is available on the factors that may regulate such structural changes.

**Results:**

We have used a range of biophysical and computational methods to further analyse the conformational flexibility and ligand binding of Pex5p. A new crystal form for the human Pex5p C-terminal domain (Pex5p(C)) was obtained in the presence of Sr^2+ ^ions, and the structure presents a novel conformation, distinct from all previous liganded and apo crystal structures for Pex5p(C). The difference relates to a near-rigid body movement of two halves of the molecule, and this movement is different from that required to reach a ring-like conformation upon PTS1 ligand binding. The bound Sr^2+ ^ion changes the dynamic properties of Pex5p(C) affecting its conformation, possibly by making the Sr^2+^-binding loop – located near the hinge region for the observed domain motions – more rigid.

**Conclusion:**

The current data indicate that Pex5p(C) is able to sample a range of conformational states in the absence of bound PTS1 ligand. The domain movements between various apo conformations are distinct from those involved in ligand binding, although the differences between all observed conformations so far can be characterised by the movement of the two halves of Pex5p(C) as near-rigid bodies with respect to each other.

## Background

Translocation of most matrix proteins to the lumen of the peroxisome depends on a signal assembled mechanism [1], in which substrate proteins carrying a C-terminal peroxisomal targeting signal type 1 (PTS1) tripeptide are translated on free ribosomes in the cytosol and recognised by the soluble receptor protein, Pex5p (reviewed in [2,3]). While the N-terminal half of Pex5p has been found to be intrinsically unstructured [4,5], the C-terminal domain of Pex5p, hereafter referred to as Pex5p(C), consists almost entirely of tandemly repeated helix-loop-helix tetratricopeptide repeats (TPRs) which are commonly found as mediators of protein-protein interactions (reviewed in [6]). It is this C-terminal domain that recognises PTS1-bearing translocation substrates. Subsequent to Pex5p(C) – PTS1 recognition, the receptor – cargo complex docks with other proteins at the peroxisomal membrane, is internalised, the cargo dissociated, and the receptor recycled (reviewed in [2,3,7]).

Human Pex5p(C) has been studied previously by X-ray crystallography: bound to a consensus PTS1 peptide (PDB code 1FCH, [8]), bound to a complete PTS1 cargo protein, sterol carrier 2 (SCP2), and in an unliganded state (2C0L and 2C0M respectively, [9]). The two liganded structures of Pex5p(C) are found to share an almost identical conformation but the apo-Pex5p(C) conformation is markedly different. While in PTS1-liganded structures, the TPR array of Pex5p(C) is "ring-like", a more loose "snail-like" arrangement is found in apo-Pex5p(C) [5,9].

Here, we report a new crystal form of apo-Pex5p(C), obtained in a crystallisation condition containing SrCl_2_. Refinement of the crystal structure reveals a novel, previously unobserved, conformation of Pex5p(C), further demonstrating the flexibility of the TPR array. In addition, Pex5p(C) is found to coordinate a single Sr^2+ ^ion within an inter-TPR loop, at a site that coincides with the hinge for the rigid-body movement of the two molecular halves with respect to one another. Synchrotron radiation circular dichroism spectropolarimetry (SRCD) indicates little difference in Pex5p(C) secondary structure in the presence or absence of PTS1 peptides, thus supporting the notion of rigid body conformational changes in solution upon cargo loading. The conformations of liganded and apo-Pex5p(C) and the presence of the Sr^2+ ^ion are discussed with reference to a set of molecular dynamic (MD) simulations. The physiological relevance of the Sr^2+ ^ion is questionable, as proton induced X-ray emission spectroscopy (PIXE) data demonstrate that Pex5p(C) has negligible affinity for Sr^2+ ^in solution – however, the Sr^2+ ^ion found in the crystal structure allows for a more detailed analysis of the propensity of this protein domain to explore conformational space.

## Results and discussion

### Structure and crystal packing

**Figure 1:**
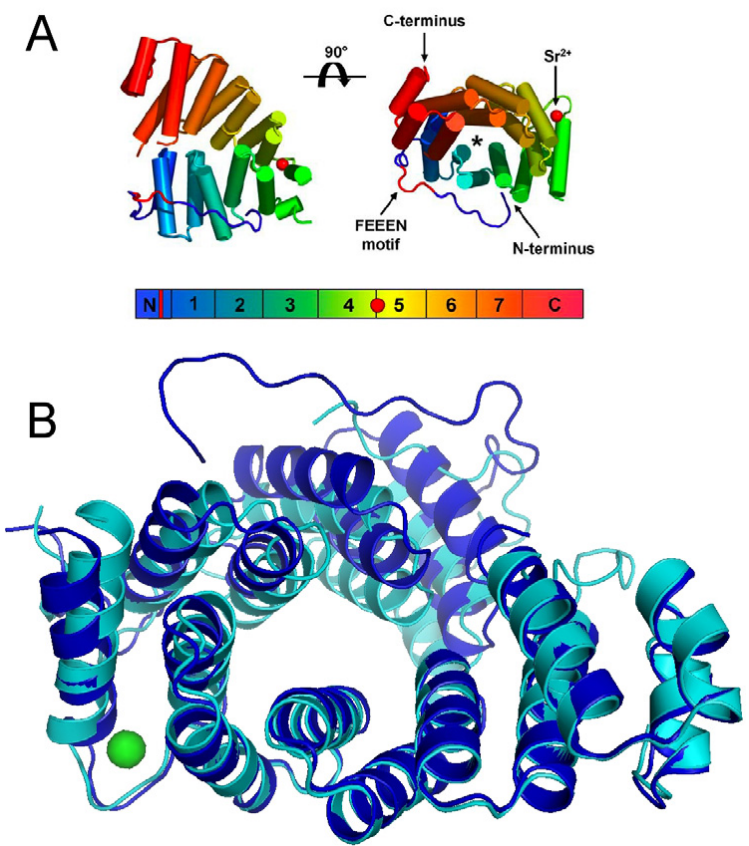
**The crystal structure of apo-Pex5p(C)**. **A. **Structure of apo-Pex5p(C) elucidated from the novel crystal form. The bar at the bottom indicates the colour progression from N- to C-terminus. The N-terminal coiled segment is indicated with an "N", the TPR motifs are numbered 1–7 and the C-terminal 3-helix bundle is labelled "C". The position of the FEEEN motif is indicated by a red bar; the strontium ion with a red circle. The cylinder model above also highlights the FEEEN motif and strontium ion in red; the "*" indicates the PTS1-binding groove. **B. **Superposition of the new (blue) and old (cyan) apo-Pex5p(C) crystal structures. The C-terminal halves were superposed, to visualise the relative difference in the orientations between the N- and C-terminal halves. The Sr^2+ ^ion is indicated in green, and it is clear that the hinge region for the observed movement lies in close vicinity to the Sr^2+^-binding site.

The structure of apo-Pex5p(C) in the new crystal form could not be easily solved by molecular replacement using our earlier structure [9] as a model, giving the first indication of a conformational difference between the two crystal forms. However, structure solution was successful when using the two halves of Pex5p(C) as models separately (Table 1, Figure 1A). The final model, containing two Pex5p(C) molecules in the asymmetric unit, indicates that the main difference between the new structure and the previous apo-Pex5p(C) is a rigid-body movement of the two halves of the molecule with respect to each other, the hinge region being located around residue Ser485 (Figure 1B).

**Table 1:** X-ray data collection and refinement statistics

Data collection	
space group	P2_1_2_1_2_1_
unit cell dimensions (Å)	53.7, 91.4, 119.9
resolution (Å)	20-2.65 (2.8–2.65)
<I/σ I>	11.7 (2.5)
R_sym _(%)	10.1 (58.1)
completeness (%)	93.5 (95.3)
redundancy	3.8 (3.8)
Refinement	
R_cryst_/R_free _(%)	24.7/31.0
rmsd bond legth (Å)	0.006
rmsd bond angle (°)	0.9
rmsd B factors (Å^2^) of bonded atoms	
main chain	0.3
side chain	0.6
average B factors (Å^2^)	
monomer A, B	44, 72
Sr ions	41
water	24

As in the previous apo-Pex5p(C) structure [9], the N-terminal half of the TPR domain is less ordered than the C-terminal region. In essence, out of all protein molecules in the Pex5p(C) crystals, 50 % have their N-terminal half very disordered; in the present crystal form, this affects one molecule in the asymmetric unit and in the previous form, two.

For both apo-Pex5p(C) crystal forms, 50 % of the protein molecules are well defined in electron density, except for a gap containing a part of the TPR4 segment. In the remaining Pex5p(C) molecules, the TPR segments 5–7 and the C-terminal helical bundle are well defined, whereas the N-terminal TPR domains 1–3 could only be modeled approximately. There is, however, no evidence to support the unfolding of considerable parts of the TPR segments. Previous [10] and current (see below) circular dichroism data show that the secondary structure contents of both the liganded and unliganded receptor conformations are almost identical. The highly flexible arrangement of TPR segments 1–3 in every other Pex5p(C) molecule found in both of the apo-Pex5p(C) crystal forms suggests that there may be even more conformational freedom than that observed in the available crystal structures.

The maximum difference in atomic coordinates between the two apo-structures is in the range of 10 Å, which is of approximately the same magnitude as seen between the apo- and liganded structures [9]. A movie made from the two conformations (additional file 1) shows that the difference seems to be caused by a sliding movement between the two halves, without any significant closing motions.

The two Pex5p(C) monomers in the asymmetric unit are related by a dyad axis. The total buried surface area, calculated by subtracting the accessible surface area of the dimer from the summed accessible areas of the monomers, is 2900 Å^2 ^at the interface; this is by far the largest area contact within the crystal lattice. Interestingly, the previous apo crystal form also shows the packing of such apparent dimers within the crystal lattice, while the rest of the crystal packing is completely different. The Sr^2+^-binding loop points towards neighbouring molecules, but the Sr^2+ ^ion is not directly involved in crystal contacts. It is possible that in addition to the hinge movement centered around the Sr^2+ ^ion binding site (Figure 1B), the rigidification of the loop (the B factors of the Sr^2+ ^ion binding loop in the B monomer are among the lowest in the entire structure) plays a role in the formation of the new crystal form.

### Comparison with previous Pex5p(C) crystal structures

Analysis of domain motions between the currently available Pex5p(C) structures using the program DynDom [11] demonstrates that, regardless of whether the PTS1 ligand is the YQSKL consensus peptide [8] or SCP2 protein [9], no potential motions can be found in liganded Pex5p(C), and the overall structure remains essentially invariant, with a whole protein backbone root mean square deviation (RMSD) best fit of 2.07 Å^2^. However, analysis of Pex5p(C) loaded with SCP2 [9] with apo-Pex5p(C) reveals significant motions, summarised in Table 2. Motions between holo-Pex5p(C) and both apo-Pex5p(C) conformations are, overall, rather similar, with whole protein backbone RMSDs of 3.06 and 3.26 Å^2^, residues 337–518/523 comprising the fixed rigid body (the whole segment of Pex5p(C) from the start of TPR1 to the end of TPR5). A rotation of the mobile rigid body (TPR6 to the C-terminus of Pex5p(C)) of approximately 20°, centered around the loop connecting TPR5 with TPR6, is observed with respect to the fixed rigid body. A significant closure motion of 80–90%, as reported by DynDom, occurs between either apo-Pex5p(C) to holo-Pex5p(C), representing the previously described ''snail-like'' to ''ring-like'' conformational change [9]. However, all of these motional similarities between the two apo-Pex5p(C) with holo-Pex5p(C) do not exclude domain motions between the two apo-Pex5p(C) states (see Table 2). The closure motion between the two apo-states is absent; nonetheless, an interdomain twist of 14.2°, centered at TPR5 is observed. Thus, both apo-Pex5p(C) conformations can be considered similarly ''snail-like'' with respect to holo-Pex5p(C), and the apparent rotation axis relating the two conformations is parallel to the line connecting the centers of mass of the rigid domains.

**Table 2:** Summary of conformational differences between PTS1-liganded and apo-Pex5p(C).

**DynDom^*a *^parameters**	**Pex5p(C)/mSCP2^*b *^*versus *previous apo-Pex5p(C)^*c*^**	**Pex5p(C)/mSCP2^*b *^*versus *new apo-Pex5p(C)**^*d*^	**new apo-Pex5p(C)^*d *^*versus *previous apo-Pex5p(C)**^*c*^
**Whole protein residues**	335–639	335–639	315–639
**Whole protein RMSD (Å^2^)**	3.06	3.26	2.20
**Fixed domain residues**	337–523	337–518	323–485
**Fixed domain RMSD (Å^2^)**	1.40	2.32	0.71
**Rotation angle (°)**	19.7	20.0	14.2
**Translation along axis (Å)**	-0.7	-0.1	0.1
**Bending residues**	495–500 (end of TPR5 helix A) 523–524 (start of TPR6 helix A) 533–537 (end of TPR6 helix A)	519–520 (loop connection TPRs 5 and 6) 530–531 (middle of TPR6 helix A) 535–536 (end of TPR6 helix A) 588–601 (entire 7C-loop)	485–489 (loop connecting TPRs 4 and 5) 496–499 (end of TPR5 helix A)
**% Closure motion**	92.0	83.8	1.7

^*a *^As described in [11] and [47]

^*b *^As described in [9], PDB indentifier 2C0L.

^*c *^As described in [9], PDB indentifier 2C0M.

^*d *^As described in this study, PDB identifier 2J9Q.

**Figure 2:**
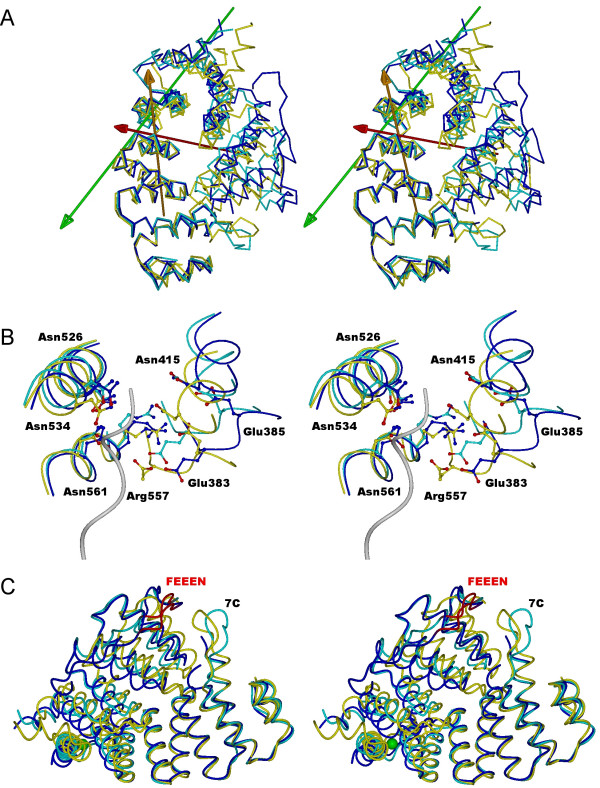
**Comparison of the new structure and previously determined Pex5p(C) structures**. **A. **Superposition of the new (blue) and old (cyan) apo-Pex5p(C) structures and the liganded structure (yellow). The arrows represent the rotation axes suggested by DynDom to describe the dynamic domain movements in the molecule. The green axis corresponds to the transition between the two apo forms. The red axis relates the new apo structure to the liganded structure and the orange one the old apo structure to the liganded structure. **B. **Disruption of the PTS1 binding site in the apo structures, caused by the movement away of the N-terminal half-molecule. The new apo structure is in blue, the old apo structure in cyan, and the liganded structure in yellow. The C-terminus of SCP2 is shown in gray, entering the binding site. In the apo structures, for example, the salt bridge between Arg557 from the C-terminal half (TPR7) and Glu residues 383 and 385 from the N-terminal half (TPR2) is broken. The positions of the 4 asparagine residues that intimately interact with the PTS1 motif are also shown. **C. **Comparison of the FEEEN motif and the 7C loop in the new apo-Pex5p(C) conformation (blue) with the previously described apo-structure (cyan) and SCP2-bound (yellow) structure [9]. The Sr^2+ ^ion in the new structure is indicated as a green sphere, and the FEEEN motif in red. The position of the 7C loop is also given.

From the above results, it can be concluded that a similar movement is required for going from both apo conformations to the liganded conformation. On the other hand, the rotation relating the two apo structures is completely different. These results are visualised in Figure 2A*via *the principal dynamic rotation axes, as reported by DynDom. The slightly different directions of closure motions required for each apo-Pex5p(C) state to accommodate the PTS1 ligand (i.e. to go from the flexible "snail-like" conformation to the rigidly defined "ring-like" conformation) suggest that Pex5p(C) exists in a pre-existing equilibrium set of conformations allowing sufficient accessibility for PTS1 binding.

At the PTS1-binding site, the rigid-body movement between the liganded conformation and either of the two apo structures disrupts central interactions and pulls apart residues involved in PTS1 binding (Figure 2B). Essentially, the tight cavity surrounding the PTS1 motif is opened up in the apo forms. Diagnostic to the conformational change is the disruption of the salt bridge formed between Arg557 of TPR7 and Glu383/Glu385 of TPR2.

Examining superimpositions of all available Pex5p(C) structures reveals interesting features in the location of the 7C-loop and the N-terminal coiled segment (Figure 2C). The 7C-loop, between TPR7 and the C-terminal helical bundle of Pex5p(C), has previously been demonstrated to be a determinant in efficient Pex5p-mediated translocation of PTS1 proteins [9,12]. This 7C-loop is clearly resolved in two of the reported structures, Pex5p(C) in complex with SCP2 (2C0L) and apo-Pex5p(C) (2C0M) [9] but is not visible in the new apo-Pex5p(C) structure presented here or in the complex with the consensus PTS1 peptide [8]. The position of the 7C-loop with the coiled segment at the N-terminus of Pex5p(C) is notably different in the presence of the SCP2 ligand (Figure 2C), with particular regard to its proximity with a highly taxonomically conserved (data not shown) acidic motif, Phe326-Glu327-Glu328-Glu329-Asn330, here designated the "FEEEN motif". The FEEEN motif is visible in all structures except the Pex5p(C)/SCP2 complex. While the FEEEN motif essentially contacts the 7C-loop in the liganded Pex5p(C) structures – in which the overall ring-like conformation is present – the FEEEN motif and 7C-loop are pulled apart in the apo-Pex5p structures, to a distance of about 15 Å (Figure 2C). This feature can most clearly be seen in the previous apo-structure (2C0M), the one available structure in which both the 7C-loop and the FEEEN motif are present. The preceding segment N-terminal to the FEEEN motif (from Leu315 onwards) occupies a different position on the surface of Pex5p(C) when the protein is PTS1-loaded (Figure 2C). It is possible that the drawing together of the FEEEN motif and 7C-loop as a result of PTS1-loading of Pex5p(C) conveys a signal to the N-terminal intrinsically unstructured [4] domain of Pex5p, stimulating downstream folding/modification/interactions of Pex5p, for example, binding with proteins of the peroxisomal membrane docking complex, such as Pex14p, which has been shown to display a higher binding affinity for cargo-loaded Pex5p than apo-Pex5p [13]. To our knowledge, *in vivo *analysis of the function of the FEEEN motif is yet to be conducted.

### Comparison with Pex5p(C) in solution

**Figure 3:**
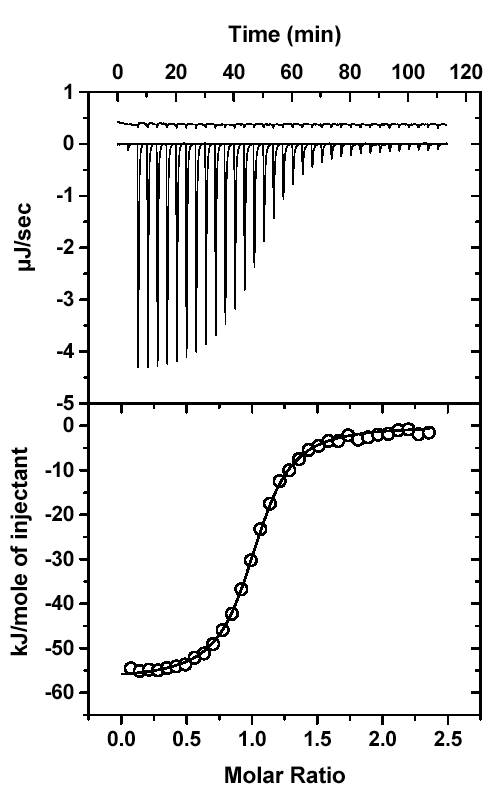
ITC analysis of the binding of the PTS1-containing peptide YQSKL to Pex5p(C). The upper panels show the raw titration data with ligand heats of dilution offset by 0.4 μJ/sec for clarity. The lower panels show the integrated enthalpies fitted to a single set of ligand binding sites in Origin 5.0.

A hexapeptide derived from the C-terminus of human SCP2, PGNAKL, has previously been demonstrated to bind to Pex5p(C) with K_*d *_= 664 ± 37 nM using ITC [9]. In the same assay, the consensus peptide YQSKL, previously co-crystallised with Pex5p(C) [8], was found to bind with K_*d *_= 429 ± 12 nM (Figure 3). Thermodynamic parameters governing the interactions of these two peptides with Pex5p(C) are shown in Table 3. Both peptides bind to Pex5p(C) with 1:1 stoichiometry. For both peptides, binding is exothermic and enthalpy driven – the negative entropy component (presumably indicative of the loss of conformational freedom of the peptide, Pex5p(C), or both, upon binding) is outweighed by the evolved heat, to give a favourable Gibbs free energy for spontaneous binding. Binding affinities of a series of variations in the YQSKL peptide have previously been intensively tested by a fluorescence anisotropy assay [8,14,15], revealing that YQSKL has a K_*d *_value in the range of 70 ± 20 nM to 250 ± 80 nM and that the variant sequence YQAKL has a slightly lower binding affinity. These results are in reasonable agreement with results presented here, and discrepancies are likely to be related to the fidelity and conditions of the assays used.

**Table 3:** Thermodynamic parameters governing PTS1 peptide binding to Pex5p(C).

**peptide**	**n**^*b*^	**ΔH (kJ/mol)**	**-TΔS (kJ/mol)**	**ΔG(kJ/mol)**	**K_d _(nM)**
**PGNAKL^a^**	1.00	-45.1	8.7	-36.4	664 ± 37
**YQSKL**	1.00	-63.1	25.5	-37.6	429 ± 12

^*a *^Data from [9].

^*b *^**n **= stoichiometry

**Figure 4:**
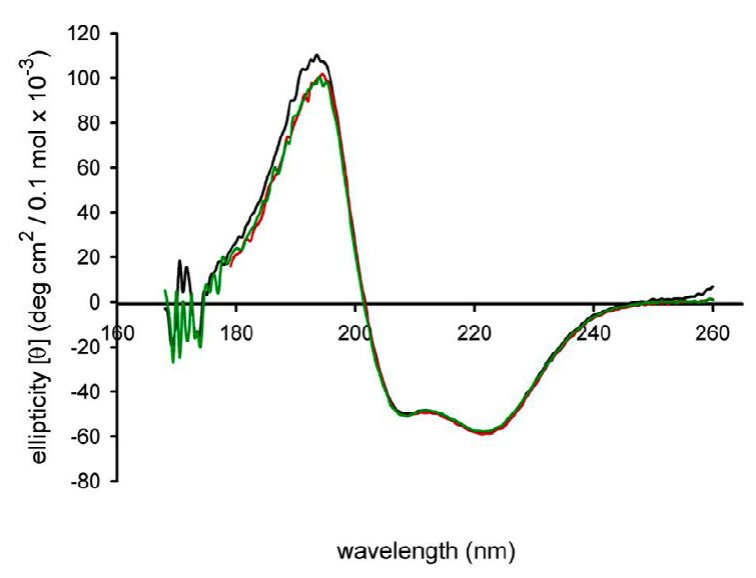
Conformation of liganded and apo-Pex5p(C) in solution. Shown are SRCD spectra of apo-Pex5p(C) (shown in black), Pex5p(C) in the presence of the consensus PTS1 peptide, YQSKL (red), and the SCP2-derived PTS1 peptide, PGNAKL (green).

Apo-Pex5p(C) was examined in solution by SRCD and compared to Pex5p(C) saturated with synthetic PTS1 peptides (Figure 4). As noted above, the two available liganded Pex5p(C) crystal structures [8,9] have almost identical conformation, regardless of whether the ligand is the consensus peptide or the SCP2 protein. SCRD spectra (Figure 4) for Pex5p(C) in the presence of the two peptides are virtually identical, with a helical content of 66.5 ± 2.2 %, as estimated by SELCON [16,17]. This value compares well with the helical content of the Pex5p(C) in complex with the consensus peptide (Chain A of PDB 1FCH, [8]), determined as 68.5 % by XTLSSTR [18]. Taken together with previous data [10], this indicates that the structure of Pex5p(C) in solution is very similar to that of crystalline Pex5p(C). SRCD spectra of Pex5p(C) liganded with PTS1 peptides and apo-Pex5p(C) (Figure 4) demonstrate that at the secondary structure level, liganded and apo-Pex5p(C) are almost identical. Two features of the α-helical spectrum result from π → π* exciton splitting – the minimum at ~208 nm (which is identical for apo- and holo-Pex5p(C)) (Figure 4) results from polarization parallel to the helical axis, while the maximum at ~190 nm results from polarization perpendicular to the helical axis [19]. Thus, the small differences in the spectra around 190 nm may indicate subtle differences in the α-helical structure arrangement. In summary, all our data are compatible with a model in which the two halves of Pex5p(C) are mobile, as (near-) rigid bodies, with respect to one another, while at the same time, secondary structure content is not significantly altered between the different conformations.

### The Sr^2+ ^ion binding site

**Figure 5:**
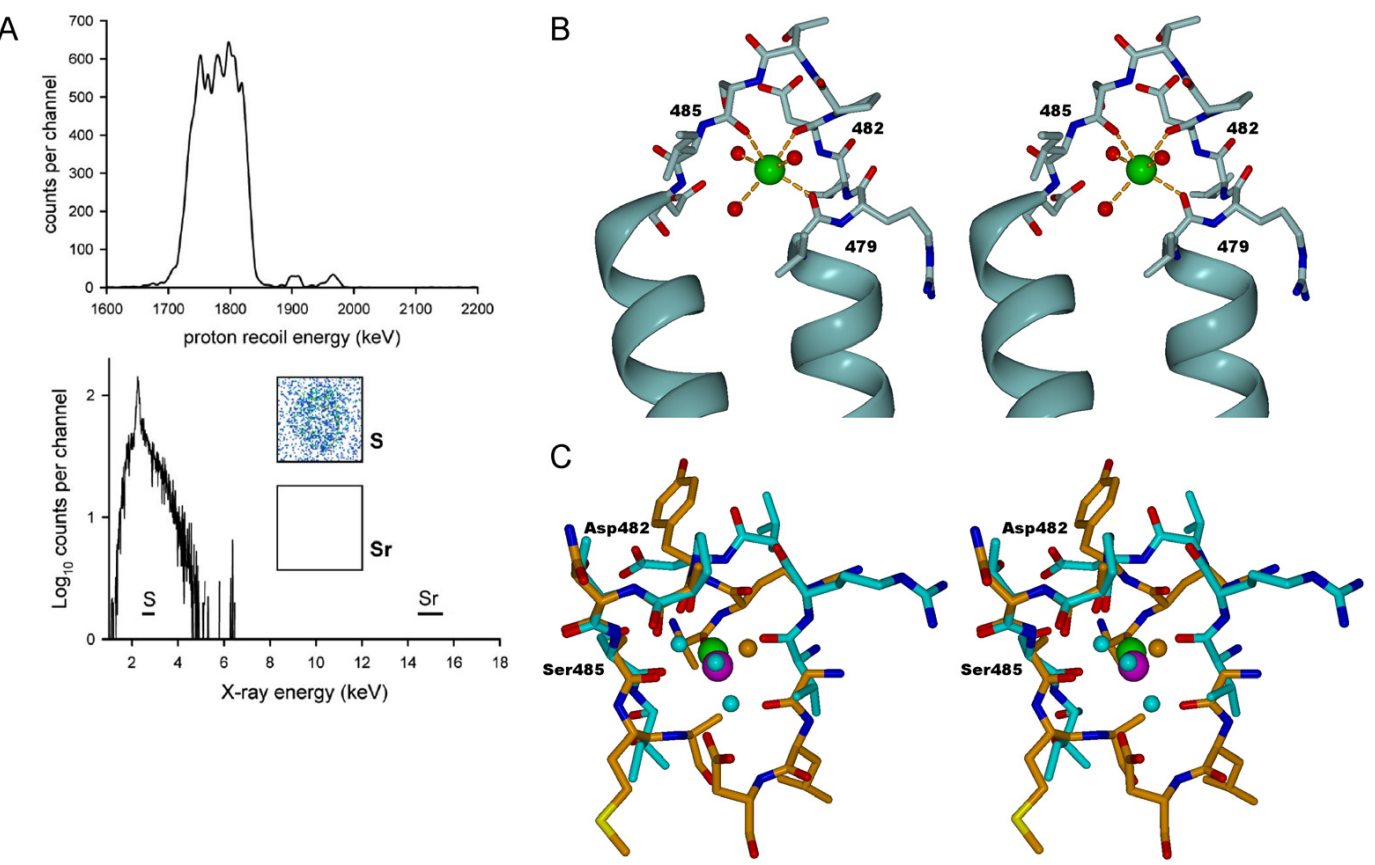
**Details of Sr^2+ ^binding by Pex5p(C)**. **A. **Elemental analysis of Pex5p(C) – the upper panel shows the Rutherford backscattering spectrum used for determining the gross matrix composition and thickness of the sample. The lower panel shows the PIXE spectrum, horizontal bars indicating the characteristic emission bands for sulphur (which can be seen) and strontium (where there are no counts). The insets compare the sulphur and strontium elemental PIXE maps from the drop of protein. **B. **Stereoview of the Sr^2+ ^binding site in the crystal structure. Residues 479–486 are shown as sticks, the backbone oxygen atoms of Val479, Asp482 and Ser485 coordinating the Sr^2+ ^ion. Three water molecules also visibly coordinate the Sr^2+^. **C. **A superposition of the Pex5p Sr^2+^-binding loop with the subtilisin CATMAT4 motif; the similarity was discovered searching the MSDmotif database [22]. Residues 482–485 of the loop in Pex5p(C) superimpose perfectly on the Ca-binding site of subtilisin. Pex5p is shown in cyan (carbon atoms and waters) and green (strontium) colours, and subtilisin in orange (carbon atoms and water) and magenta (calcium).

Crystallisation of Pex5p(C) clearly demonstrated a preference for strontium – no crystals could be grown in the presence of other divalent cations: magnesium, calcium, barium, cadmium, cobalt, copper, manganese, yttrium, iron, zinc, or nickel (data not shown). However, upon incubation in an excess of strontium in solution and subsequent removal of free strontium by dialysis, no specific binding of the metal could be detected – an elemental analysis by PIXE demonstrated the presence of a maximum of 5 × 10^-5 ^strontium ions per molecular of protein, calibrated against the X-ray fluorescence signal from sulphur atoms present in Pex5p(C) (Figure 5A).

The strong electron density found in the crystal structure reveals that the single Sr^2+ ^ion is coordinated by the backbone oxygen atoms of three Pex5p(C) residues – Val479 (2.52 Å), Asp482 (2.46 Å), and Ser485 (2.53 Å) – and by three water molecules, at Sr-O distances of 2.36, 2.47, and 2.81 Å (Figure 5B). Examination of other Sr^2+ ^ion binding proteins deposited in the PDB [20,21] does not suggest any clearly similar Sr^2+ ^ion binding sites, although in general, a similar coordination system, forming a tetragonal bipyramid, can be observed.

On the other hand, a search for small 3D motifs at the EBI MSDMotif server [22] indicates a large number of Ca^2+ ^ion binding sites found in various proteins that have a similar loop backbone conformation as seen in the Sr^2+ ^ion binding loop; the motif is called CATMAT4 in the database. One of them, that from subtilisin determined at 0.78-Å resolution [23], is shown in figure 5C for comparison. While it is possible that this loop in Pex5p(C) can bind divalent cations at low affinity, no physiological relevance for such binding can be established at the moment. The binding of Sr^2+ ^ion to the loop probably stabilises the conformation of Pex5p(C) seen in our new crystal form.

### Molecular dynamics simulations of apo-Pex5p(C)

**Figure 6:**
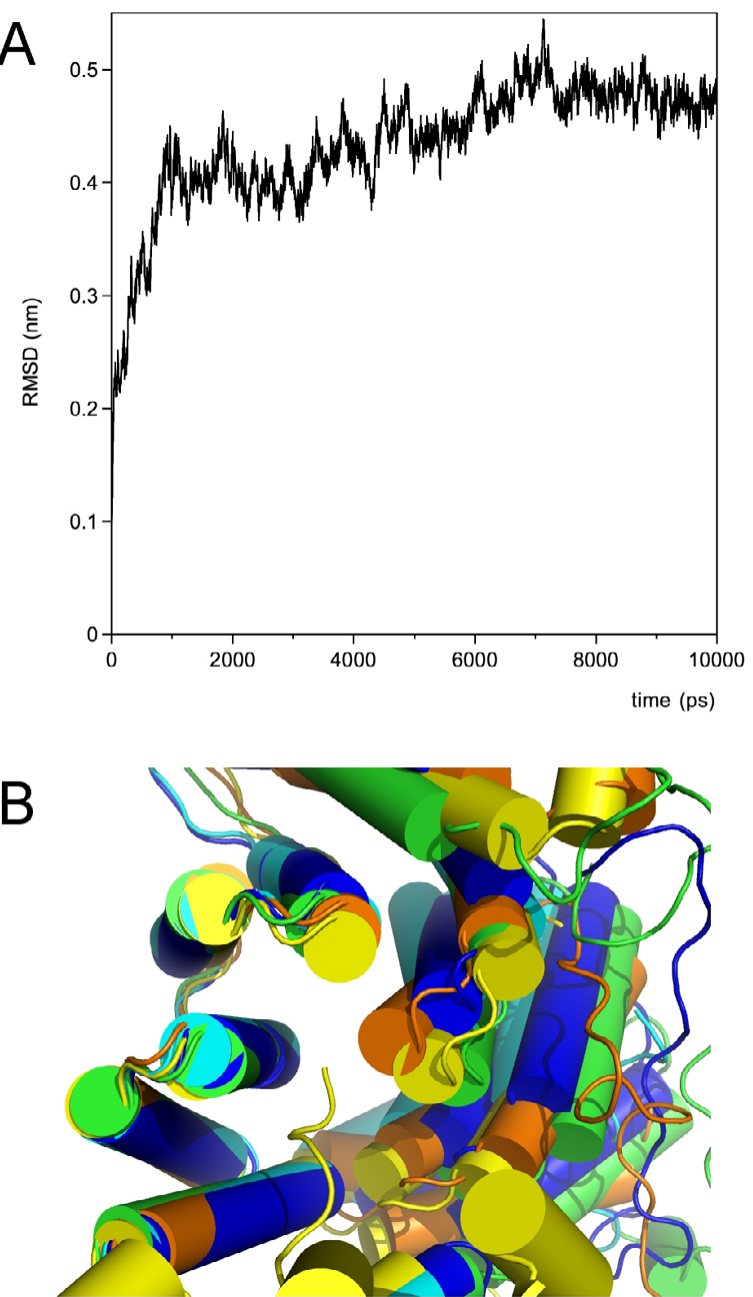
**Molecular dynamics simulations in the presence and absence of Sr**. **A. **A comparison between the Pex5p(C) structures along the simulation trajectories. The difference between the simulation in the presence and absence of the Sr^2+ ^ion is plotted as a function of running time. **B. **Superposition of the new apo structure (blue) with the old apo structure (cyan), the SCP2-loaded structure (yellow), and the simulation end-states in the presence (green) and absence (orange) of the Sr^2+ ^ion. Residues 503–639 were used for the superposition to highlight the orientation between the N- and C-terminal halves. The tail of SCP2 enters the binding cavity from below (yellow ribbon). Note the similarity between the liganded structure and the simulation without Sr^2+^. An animated presentation of the result can be found in the additional data file 2.

In order to elucidate the putative effect of the bound Sr^2+ ^ion on the dynamic properties of Pex5p(C), 10-ns MD simulations were carried out. The starting structure was the new crystal structure of apo-Pex5p(C) described above, and two runs were performed: with and without the Sr^2+ ^ion. An overall comparison of the trajectories by means of the RMSD between them indicates clearly that the simulations take different paths, depending on the presence of Sr^2+ ^(Figure 6A). At 1 ns, the RMSD reaches 4 Å, reaching equilibrium at approximately 4.7 Å after 6 ns.

The end-point coordinates of the MD simulations were analysed with respect to their similarity to experimental apo and liganded structures of Pex5p(C). A multiple structural alignment was carried out using SSM [24], and the resulting RMSD values for 260 Cα atoms are shown in table 4. While the Sr^2+^-loaded structure remains rather close to the starting conformation, the conformation of the Sr^2+^-depleted structure after simulation indeed most closely resembles the conformation seen in the liganded Pex5p(C) structures.

**Table 4:** RMSDs (Å) for Cα atoms based on multiple structural alignment [24] of the experimental structures and the end-point structures from the MD simulations.

	Pex5p(C)-apo1 (current)	Pex5p(C)-apo2	Pex5p(C)-SCP2	MD with Sr	MD w/o Sr
Pex5p(C)-apo1 (current)	-	2.03	2.39	1.77	2.12
Pex5p(C)-apo2		-	2.31	2.28	2.61
Pex5p(C)-SCP2			-	2.46	2.09
MD with Sr				-	2.66
MD w/o Sr					-

The above observation was confirmed by carrying out manual superposition of all structures (experimental apo and liganded structures plus the two simulation end-points), using residues 503–639 of the C-terminal half for superposing. Clearly, the removal of Sr^2+ ^prior to simulation brings the simulation end-point very close to the liganded conformation (Figure 6B). A supplementary file containing a QuickTime movie of the superposition is also available (additional file 2). In line with this, DynDom analysis of domain motions between the simulation end points and the liganded Pex5p(C) coordinates indicates that no dynamic domains are found between the liganded conformation and the simulated structure in the absence of Sr^2+^, while a closure motion of 62 % can be seen when using the simulated structure in the presence of Sr^2+^. Thus, overall, the MD simulations suggest that the PTS1-liganded conformation of Pex5p(C) is also within the conformational space that can be sampled by apo-Pex5p(C).

## Conclusion

Protein transport by the Pex5p receptor across the peroxisomal membrane requires conformational changes in the TPR domain towards its C-terminus. It is clear that such changes are instrumental for PTS1 recognition and binding, and also likely for protein cargo release into the peroxisomal lumen. In order to shed further light on the mechanism, by which conformational changes occur in Pex5p(C), we carried out extended studies on Pex5p(C) ligand binding and structure, using a variety of biophysical and theoretical methods. The results obtained complement our earlier studies on Pex5p(C) in complex with SCP2 [5,9,10], and give important new information on the structure-function relationships in Pex5p(C).

All in all, our data strongly suggest a dynamic nature for Pex5p(C), such that the two halves of the TPR domain preferably move as near-rigid bodies related by a single hinge around TPR5. The movement relating different apo conformations to each other is different from that relating the apo conformations to the liganded form. On the other hand, unliganded Pex5p(C) closes up to adopt a "nearly liganded" conformation in MD simulations, while the one having a single Sr^2+ ^ion bound does not. This indicates a role for the Sr^2+^-binding loop in Pex5p(C) dynamics, although Sr^2+ ^is unlikely to be a physiologically relevant ligand. Rather, the results suggest that the more rigid conformation of the loop induced by the bound Sr^2+ ^can restrict the movement between the two lobes of the TPR domain.

Based on the current and earlier data, three regions clearly emerge as being putatively important for regulating the dynamics and ligand binding of Pex5p(C): the 7C-loop, the FEEEN motif, and the Sr^2+^-binding loop. The 7C-loop and the N-terminal region containing the FEEEN motif interact in the liganded state, but not in the apo conformations. On the other hand, the hinge for rigid body movement between the N- and C-terminal halves of Pex5p(C) lies in close proximity of the Sr^2+^-binding site seen in the present crystal structure. It is possible that the interactions of these regions with other molecules in or near the peroxisomal membrane affect the ability of Pex5p to bind and release its cargo properly.

To conclude, Pex5p(C) is able to adopt distinctly different conformations in its apo state. These conformations do not involve changes in secondary structure content, but can be mainly described as rigid-body movements about a single hinge, located at the Sr^2+ ^binding loop and the following helix of TPR5. Furthermore, PTS1 ligand binding also occurs *via *near-rigid body movement of the two halves of Pex5p(C), without detectable change in secondary structure content. The origin (or hinge) for the latter movement lies in the TPR5-6 segment. As can be seen from our Sr^2+^-liganded structure, it is possible that the binding of a ligand to the region comprising TPR domains 4–6 can affect the overall conformation of Pex5p(C), further changing its functional properties.

## Methods

### Crystallisation and data collection

Preparation of recombinant Pex5p(C), representing the C-terminal TPR domain of the long isoform of human Pex5p (residues 315 – 639) has been previously described [9]. Prior to crystallization, Pex5p(C) was dialysed against 20 mM bis-Tris-propane, 20 mM KCl, 1 mM Tris-(2-carboxyethyl)-phosphine hydrochloride (pH 7.0) and concentrated to 8 mg/ml by centrifugal ultrafiltration. Crystals were obtained by mixing 1 μl protein with 1 μl reservoir solution, using the hanging drop vapour diffusion method at 277 K. Reservoir buffer conditions were optimised to 23% (w/v) PEG 3350, 100 mM Tris-HCl (pH 8.75) and 10 mM SrCl_2_. Crystals grew within 10 days.

X-ray diffraction data were collected at the EMBL-Hamburg synchrotron beamline X13 (DESY, Hamburg, Germany). Data were obtained from a single crystal captured in a fibre loop and directly cooled to 100 K in a stream of gaseous nitrogen. Data were processed and scaled using XDS [25] and XDSi [26]. For cross-validation of subsequent steps, 5% of the data were randomly selected and not used in refinement. X-ray data collection statistics are summarised in Table 1.

### Structure solution, refinement, and analysis

The structure was solved by molecular replacement using MOLREP [27]. No solution was found when using the whole apo-Pex5p(C) domain as model, even though 2 molecules were expected to be present in the asymmetric unit. This was a clear indication of a difference in conformation between apo-Pex5p(C) in the current and previous crystal form [9]. The correct solution was found step by step, by dividing the search model into two halves and searching for the halves separately, with intermediate refinement and rebuilding. For both molecules in the asymmetric unit, the C-terminal half (residues 466 to 639) was found first, followed by the N-terminal half (residues 315 to 442). Refinement was carried out using REFMAC5 [28], and model building was performed in COOT [29]. When the built model was nearly complete, TLS parameters [30] were employed. TLS groups and parameters suggested by the TLSMD server [31] were also used during refinement. The refinement statistics are listed in table 1. The final refined model, as well as the structure factors, have been deposited at the Protein Data bank and have accession code 2J9Q.

Structure comparison was carried out using DynDom [11], MSDMotif [22], SSM [24], and O [32]. Figures and movies were made using DINO [33], PyMOL [34], POV-Ray [35], and the Morph server [36,37].

### Isothermal titration microcalorimetry (ITC)

Binding of a synthetic consensus PTS1 peptide YQSKL [8] to Pex5p(C) was analysed by ITC, as previously described for PGNAKL (representing the C-terminus of PTS1 protein SCP2 [9]). Data were fitted using Origin 5.0 (MicroCal, Northampton, MA, USA).

### Synchrotron radiation circular dichroism (SRCD)

Pex5p(C) was dialysed against 10 mM potassium phosphate (pH 7.4) and diluted to 1 mg/ml. When appropriate, samples were mixed with a 10-fold molar excess of synthetic PTS1 peptides (Sigma-Genosys, UK) YQSKL or PGNAKL. Spectra were measured on SRS beamline station CD12 (CCLRC Daresbury, UK) [38] and data analysed as described [10]. Peptide and buffer background were subtracted at the outset of data analysis. Secondary structure estimations from SRCD spectra were made using the SELCON software [16,17] and defined from crystal structures using XTLSSTR [18].

### Proton induced X-ray emission (PIXE)

Pex5p(C) at 80 μM(~3 mg/ml) was incubated with 1 mM SrCl_2 _for 3 hours at room temperature and subsequently exhaustively dialysed against 200 mM ammonium acetate (pH 7.4) and diluted to 2.1 mg/ml. 0.2 μl samples were dried on to mylar films of 2 μm thickness. Elemental analysis was conducted at the National Ion Beam Centre, University of Surrey, UK, using a 2.5-MeV proton beam of 3-μm diameter [39]. X-ray emission and Rutherford backscattered proton spectra were measured as previously described [40], and the data were analysed using the GUPIX software [41].

Briefly, the Rutherford backscattering proton spectra were analysed to give the CNO composition of the matrix and its thickness, so the self absorption of the X-rays could be corrected for. All the other quantitative information comes from the X-ray spectra. The elemental maps were made from scanning the proton beam in x and y directions and collecting X-ray spectra at each point, setting a software gate around the sulphur and strontium peak. Then, the number of X-rays in the peak at that x-y position were sorted into a 2-D plot.

### Molecular dynamics simulations

As a starting point, the new crystal structure of Sr^2+^-bound apo-Pex5p(C) was used. An energy minimisation and a 10-ns molecular dynamics (MD) simulation of the free protein in water was performed with the Gromacs 3.2.1 package, both with and without a Sr^2+ ^ion [42,43], using the OPLS-AA/L force field. The simulation box corresponded to a truncated dodecahedron, containing the protein, single point charge water molecules [44], and Na^+ ^ions to neutralize the system. The box size was determined by setting the distance between the protein and the box to 0.6 nm. Dummy hydrogen atoms were used. Weak temperature coupling (300 K) and pressure coupling (1.0 bar) were utilized [45] with coupling constants of 1.0 ps. Coulomb interactions were computed with the fast particle mesh Ewald summation (PME) with a grid spacing of 0.12 nm and fourth-order interpolation. The LINCS algorithm was employed to constrain bonds [46]. The centre of mass motion was removed on every step. The system was energy-minimised, followed by a short (10 ps) position restraining run for further equilibation. The 10-ns simulation was performed using the Brutus Cluster at the University of Oulu, Finland.

## Abbreviations

ITC isothermal titration microcalorimetry

PIXE proton induced X-ray emission spectroscopy

PTS1 peroxisomal targeting signal type 1

SCP2 sterol carrier protein 2

SRCD synchrotron radiation circular dichroism spectropolarimetry

TPR tetratricopeptide repeat

Pex5p(C) C-terminal domain of human peroxin 5, the PTS1 receptor

RMSD root mean square deviation

## Authors' contributions

WAS conceived of the study together with MW and PK, cloned and purified the protein, and carried out ITC, SRCD, crystallisation, diffraction data collection, and data analysis. NP and AJ carried out MD simulations, and EFG carried out the PIXE analysis. PK solved the crystal structure and carried out structure analysis. All authors participated in drafting the manuscript and read and approved the final manuscript.

## Supplementary Material

Additional file 1 Morphing movie between the two experimentally determined conformations of apo-Pex5p(C). An animation of the difference between the two apo conformations of apo-Pex5p(C). The molecule is coloured from the N-terminus (blue) to the C-terminus (red). The predicted motion has a sliding nature instead of being a closing motion. Click here for file

Additional file 2 A superposition of the experimental and simulated structures of Pex5p(C). This is an animated presentation of figure 6B. See legend for figure 6B for additional details. Click here for file
