# Supplementary figures and images for: A previously unobserved conformation for the human Pex5p receptor suggests roles for intrinsic flexibility and rigid domain motions in ligand binding
Source: BMC Struct Biol. 2007 Apr 11;7:24. doi: 10.1186/1472-6807-7-24 (PMC1854907; doi:10.1186/1472-6807-7-24)

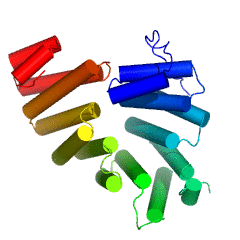

Supplement: Additional file 1 — Morphing movie between the two experimentally determined conformations of apo-Pex5p(C). An animation of the difference between the two apo conformations of apo-Pex5p(C). The molecule is coloured from the N-terminus (blue) to the C-terminus (red). The predicted motion has a sliding nature instead of being a closing motion. [file 1472-6807-7-24-S1.gif]
